# The standard map: From Boltzmann-Gibbs statistics to Tsallis statistics

**DOI:** 10.1038/srep23644

**Published:** 2016-03-23

**Authors:** Ugur Tirnakli, Ernesto P. Borges

**Affiliations:** 1 Department of Physics, Faculty of Science, Ege University, 35100 Izmir, Turkey; 2 Instituto de Fìsica, Universidade Federal da Bahia, Rua Barão de Jeremoabo, 40170-115 Salvador-BA, Brasil; 3 National Institute of Science and Technology of Complex Systems, Rua Xavier Sigaud 150, 22290-180, Rio de Janeiro - RJ, Brazil

## Abstract

As well known, Boltzmann-Gibbs statistics is the correct way of thermostatistically approaching ergodic systems. On the other hand, nontrivial ergodicity breakdown and strong correlations typically drag the system into out-of-equilibrium states where Boltzmann-Gibbs statistics fails. For a wide class of such systems, it has been shown in recent years that the correct approach is to use Tsallis statistics instead. Here we show how the dynamics of the paradigmatic conservative (area-preserving) stan-dard map exhibits, in an exceptionally clear manner, the crossing from one statistics to the other. Our results unambiguously illustrate the domains of validity of both Boltzmann-Gibbs and Tsallis statistical distributions. Since various important physical systems from particle confinement in magnetic traps to autoionization of molecular Rydberg states, through particle dynamics in accelerators and comet dynamics, can be reduced to the standard map, our results are expected to enlighten and enable an improved interpretation of diverse experimental and observational results.

Exponential and Gaussian distributions are signatures of the Boltzmann-Gibbs statistical mechanics. These distributions are those that maximise the Boltzmann-Gibbs entropy and ensure the equilibrium state. The Maxwell distribution is an instance of the equilibrium distribution for the velocities of molecules in an ideal gas. The underlying mathematical reason for this is the existence of the standard Central Limit Theorem (CLT)^1^. On the other hand, due to ergodicity breaking, some systems remain indefinitely trapped into non-exponential and non-Gaussian distributions, and thus achieve out-of-equilibrium quasi-stationary states. The *q*-exponential and the *q*-Gaussian distributions are functions associated with some of these quasi-stationary states and they are the maximising distributions for the non-additive Tsallis entropy given by^2^


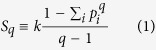


where Boltzmann-Gibbs entropy is a special case as *q* → 1. This feature permits to describe these special non-equilibrium states with the same formal framework of the equilibrium thermostatistics, known as Tsallis statistics^3^, and this general picture is reduced to the equilibrium one if the parameter *q* attains a special limiting value (*q* → 1). In this case, the underlying mathematical mechanism is the generalized CLT^4^,^5^, which states that the stable limit distributions for a certain class of systems in such quasi-stationary states are *q*-Gaussians. Therefore, the role of *q*-Gaussians in Tsallis statistics is basically the same as that of Gaussians in Boltzmann-Gibbs statistics. In this work we show, for the first time, that these two cases coexist in the classical standard map, and discuss the necessary conditions under which one case prevails over the other one. This neatly illustrates the respective domains of validity of Boltzmann-Gibbs and of Tsallis statistics. The results are important not only from the statistical mechanics theoretical viewpoint but also due to their potential of being applicable to diverse fields of physics. Indeed, many physical systems can, as a first approximation, be reduced to the standard map. This is so for particle confinement in magnetic traps^6^, particle dynamics in accelerators^7^, comet dynamics^8^, ionization of Rydberg atoms^9^, and electron magnetotransport^10^, among others.

Non-Gaussian distributions, particularly *q*-Gaussians, have been observed in nature in several experimental, observational and model systems^3^. Impressive experimental examples include (i) a high dimensional dissipative system where the probability densities of velocity differences measured in a Couette-Taylor experiment for a fully developed turbulence regime^11^,^12^, (ii) transport properties of cold atoms in dissipative optical lattices^13^,^14^, (iii) transverse momentum spectra of hadrons at LHC experiments^15^ and (iv) confined granular matter^16^. As observational works, for small bodies in the Solar System, particularly asteroid rotation periods and diameters^17^ and distribution of meteor showers^18^ can be given. At a larger scale, the rotation curve for the M33 Triangulum Galaxy has been successfully analyzed in the same sense^19^. Among model systems, one of the paradigmatic dissipative low dimensional model, the logistic map, has been numerically investigated and *q*-Gaussians have been found as the chaos threshold is approached using the band splitting structure obeying the Huberman-Rudnick scaling law^20^,^21^,^22^.

*q*-Gaussians have also been recently observed in a conservative high dimensional model^23^. In the *α*-XY model, i.e., a system of *N* classical localized planar rotators with two-body interactions and periodic boundary conditions, the potential is assumed to decay with distance as 1/*r*^*α*^, and *α* ≥ 0 is the parameter that controls the range of the interactions, short-range for *α/d* > 1, and long-range for 0 ≤ *α*/*d* ≤ 1 (*d* is the spatial dimensionality of the system).

Recently a generalization of the conservative one-dimensional Fermi-Pasta-Ulam model, properly modified to account for linear and nonlinear long-range interactions, has been analyzed. The range of the interactions is controlled in the same way as for the *α*-XY model just mentioned. Ordinary Gaussians are observed when short-range interactions (*α* > 1) are present, and *q*-Gaussians are observed when long-range interactions (0 ≤ *α* ≤ 1) are present^24^. It has been found that the maximal Lyapunov exponent *λ* asymptotically decreases as *N*^−*κ*(*α*)^, in a rather similar way of that observed in^25^ for the *α*-XY model and the *q*-Gaussian distributions that emerge are characterized by the parameter *q* that depends on *α*.

All these systems appear to share in common the following scenario: ergodicity in a region is characterized by the largest Lyapunov exponent *λ* and two regimes shall be distinguished in the thermodynamic limit (number of particles *N* → ∞). (i) Strongly chaotic regime corresponds to a large positive Lyapunov exponent, where the system is ergodic. The dynamics of the system evolves to an equilibrium state described by Boltzmann-Gibbs statistical mechanics, with exponential or Gaussian distributions (according to the considered dynamical variable); (ii) Weakly chaotic regime corresponds to a very small positive Lyapunov exponent (*λ* ≈ 0), where the system behaves for a very long time as non-ergodic. Distributions of the dynamical variables are not exponential or Gaussians, and the Boltzmann-Gibbs framework is not suitable for this case. *q*-Gaussian distributions have been observed for this case by proper time and ensemble averages^26^. These distributions are obtained by maximisation of the nonadditive entropy *Sq*^2^,^27^,^28^ (in exactly the same manner as Gaussian distributions are obtained by maximisation of the Boltzmann-Gibbs entropy), which is a strong indication that these systems are connected to nonextensive statistical mechanics^3^. They may be written as


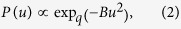


where *B* > 0 is the Lagrange parameter and the *q*-exponential is given by


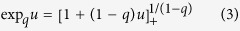


where [*A*]_+_ ≡ max {0, *A*} and its inverse, *q*-logarithm, is defined by 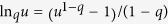. The ordinary Gaussian, exponential and logarithm functions are respectively recovered in the limit *q* → 1.

In this paper we consider the standard map, that is a paradigmatic low dimensional conservative (area-preserving) model, and we follow the averaging procedure originally used for the logistic map, as described in^20^,^21^,^22^. As will be discussed in detail below, this paradigmatic model offers an excellent medium for us to analyse both regimes explained above and to establish a connection between these regimes where the system is ergodic and non-ergodic.

## Results

**Figure 1:**
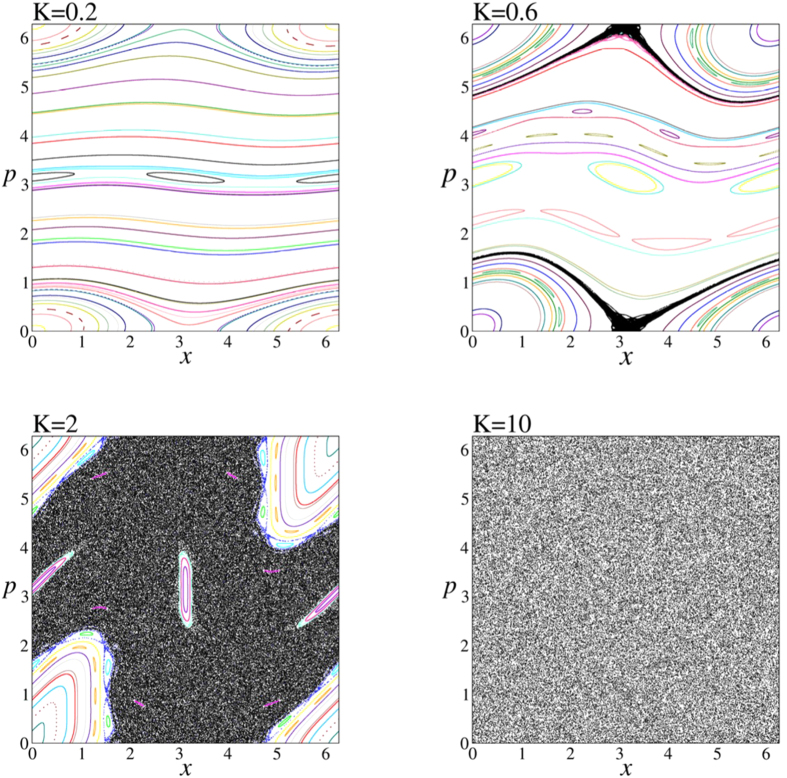
Phase portrait of the standard map for 4 representative *K* values. In each case, black dots represents the region of chaotic sea in the available phase space and all other colors represent different stability islands.

The standard map is defined as^29^,^30^,^31^


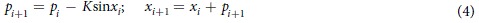


where *p* and *x* are taken as modulo 2*π*. This map has very rich properties depending on the map parameter *K*. Here, we will focus on four representative cases whose phase portraits are given in Fig. 1. The two extreme cases are *K* = 0.2 and *K* = 10, one of which represents the domination of the phase space with the stability islands and the other is clearly an example of the invasion of the full phase space by the chaotic sea. On the other hand, the other two cases in between, namely, *K* = 0.6 and *K* = 2, are good examples in order to see how these regions with stability islands and chaotic sea merge in the available phase space. It is clear that if the system starts from an initial condition located on one of the archipelagos (given by the same color), it will stay forever in the same archipelago, whereas if it starts from somewhere in the chaotic sea, the iterates will cover the whole chaotic region.

**Figure 2:**
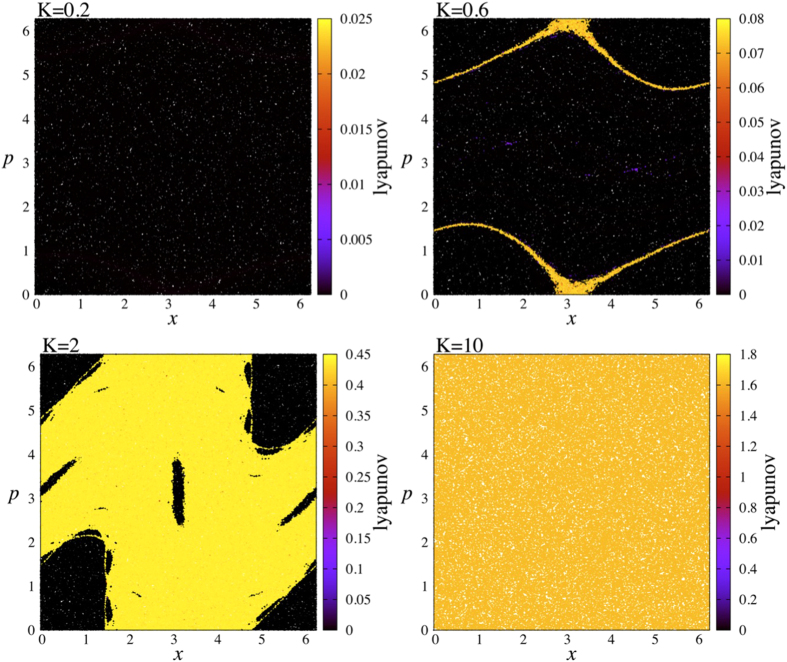
Lyapunov exponent results of the phase portrait of the standard map. The same representative *K* values are used. For each case, Lyapunov exponents are calculated for 200000 initial conditions. In the calculation, each initial condition is iterated 10^7^ times.

At this point, we need to calculate the largest Lyapunov exponent of these cases using the Benettin algorithm^32^ but this calculation is to be done very carefully. Generally, calculating the Lyapunov exponent by taking an ensemble average would not be exactly correct here since the contributions coming from the initial conditions of stability islands are much smaller than the ones coming from the chaotic sea. Therefore, making an ensemble average would not reflect the correct behaviour of the system. In order to reflect the correct behaviour, we prefer to plot the largest Lyapunov exponents as given in Fig. 2, where we calculate the exponent of each initial condition separately over the whole phase space and the magnitude of the exponents are given by a color map. The present full phase-space representation of the Lyapunov exponents of the standard map is here exhibited for the first time, to the best of our knowledge. It enables a novel and very neat understanding of the dynamical foundations of statistical mechanics. As seen in the figure, the case *K* = 0.2 represents a Lyapunov spectrum in which all results are extremely close to zero (black dots), whereas the case *K* = 10 conversely exhibits a spectrum where all results are largely positive (yellowish dots). This means that, in the former case (latter case), the whole phase space is dominated by the stability islands (chaotic sea). On the other hand, the other two cases, *K* = 0.6 and *K* = 2, are good examples where the phase space consists of both stability islands and chaotic sea. This way of representing the Lyapunov spectrum allows us to see clearly the portions of the whole phase space where the system is ergodic and non-ergodic for a given *K* value.

Now we can analyze the limit distributions of the standard map for these representative *K* values. We define the variable


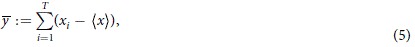


where the average 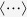 is calculated as time average taken over not only a large number of *T* iterations, but also a large number of *M* randomly chosen initial values, namely,


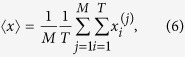


and calculate the probability distribution of 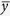, namely 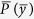, for any given *K* parameter.

**Figure 3:**
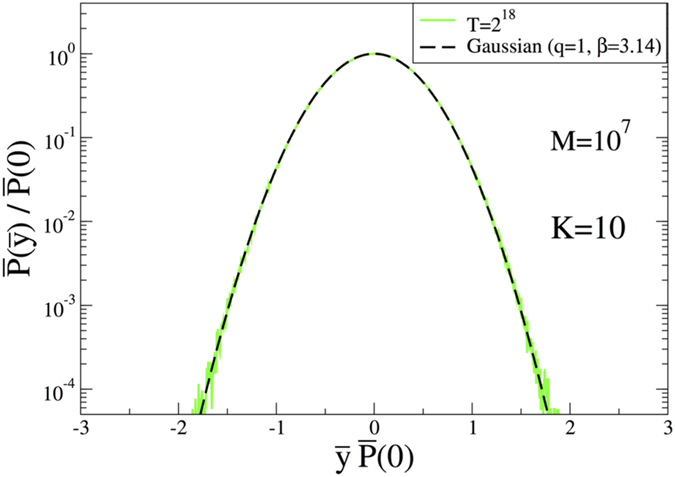
Normalized probability distribution function for the case *K* = 10 with *T* = 2^18^.

Let us start with the result of the case *K* = 10, where the probability distribution is expected to be a Gaussian since for this case the phase space is totally a chaotic sea, which makes the whole system ergodic. Therefore, in this case, taking 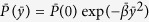 and making the transformations 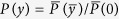, 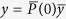 and 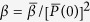, one can easily find


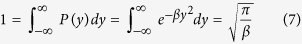


from where the value of *β* parameter of the Gaussian is obtained as *β* = *π*. The simulation result is given in Fig. 3 where a clear Gaussian with *β* = *π* value is easily seen as expected. It should also be noted that the stable limit distribution is obtained quickly. We have checked that *T* = 2^18^ is more than enough for the system to achieve the stable distribution. In all these simulations we use a large number of initial conditions (*M* ≥ 10^7^) to achieve better statistics.

**Figure 4:**
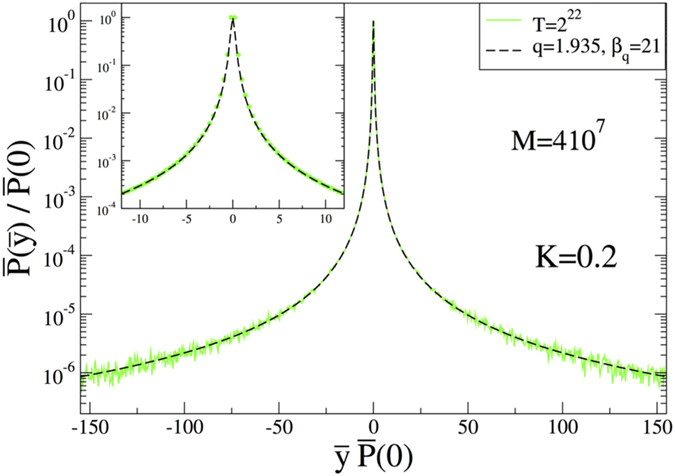
Normalized probability distribution function for the case *K* = 0.2 with *T* = 2^22^. In the Inset, the central part is zoomed for a better visualization.

**Figure 5:**
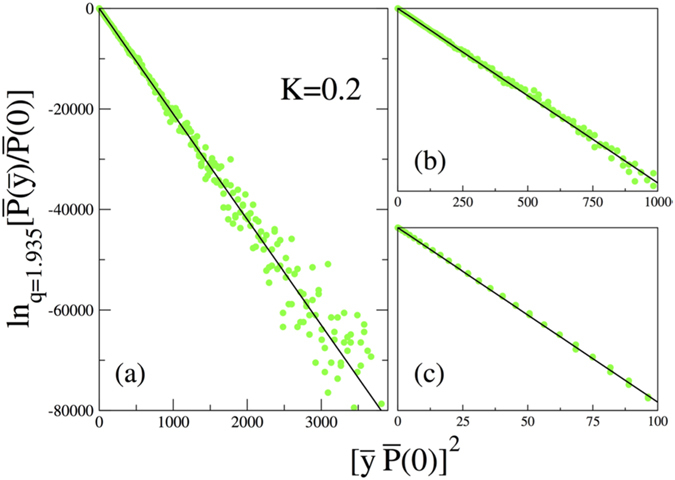
*q*-logarithmic representation of the normalized probability distribution (**a**) for the tails, (**b**) for the intermediate region and (**c**) for the central part of the case *K* = 0.2.

Now we can investigate the case *K* = 0.2, where the probability distribution is expected to be a non-Gaussian due to the change of the phase space from being totally chaotic to totally consists of stability islands, which makes the whole system non-ergodic. The result is given in Fig. 4 where, instead of a Gaussian, now a clear *q*-Gaussian is observed with *q* = 1.935. In this case, the meta-stable limit distribution happens to be achieved slowly but at the level of *T* = 2^22^ it has already been reached. We have checked it with *T* = 2^23^ and verified that the distribution does not change in the displayed region. At this point, it must be noted that, although the theoretical expectation is to find a Gaussian as a limiting distribution as *t* → ∞ since the Lyapunov exponent is very close to zero but still positive, there is no numerical evidence for this and for any practical application the only distribution that we observe is this meta-stable distribution. We also plot the same data as *q*-logarithm of the probability distribution in Fig. 5 in order to see whether it is a straight line or not. For three different regions (i.e., the region including the tails, intermediate region and the central part), the straight line is well approached. The fact that we observe straight lines in all scales excludes other distributions that are asymptotic power laws, like Lévy distributions. The underlying reason for the appearance of *q*-Gaussians can be explained in terms of the notions borrowed from one-dimensional dissipative systems (the logistic map) discussed in^20^,^21^,^22^. In general, ergodicity breaking alone cannot give rise to these distributions to appear, a special type of correlations among random variables is also needed. These conditions are fulfilled for the logistic map as chaos threshold is approached by means of Huberman-Rudnick scaling inside the band structure. The iterates inside each band of a given band structure are independent and identically distributed but all iterates are strongly correlated. In the case of the standard map, the islands in one archipelago are like bands in one band structure in the logistic map. The iterates inside one island are independent and identically distributed but as a whole all iterates in one archipelago are strongly correlated. These strong correlations happen to be in the class of correlations which yields *q*-Gaussians, and are expected to be the correlations discussed in 4 or similar ones.

**Figure 6:**
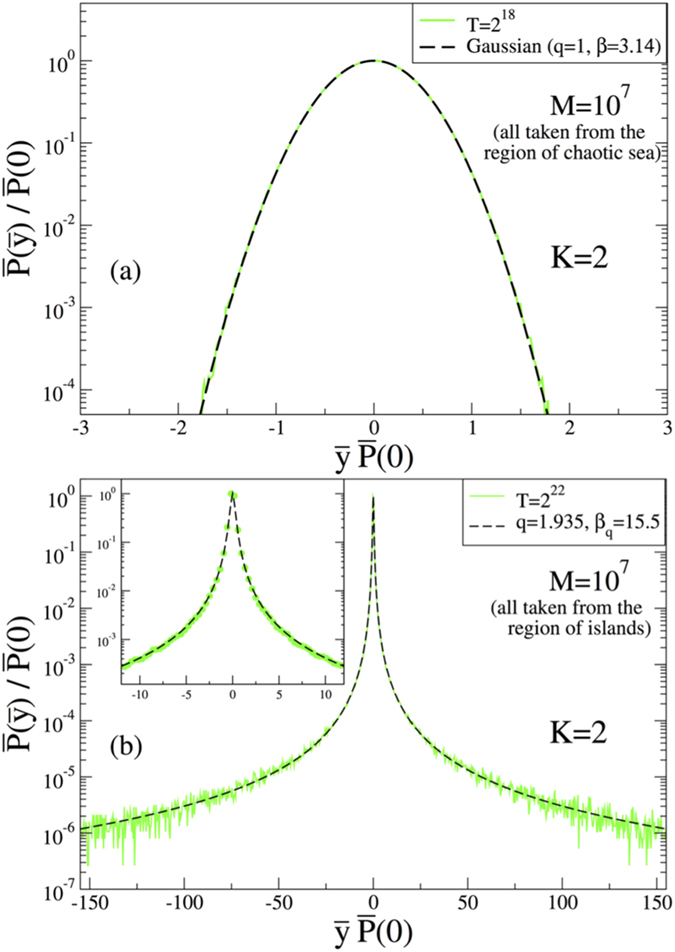
Normalized probability distribution function for the case *K* = 2. In the calculations, all initial conditions are taken from the region of (**a**) chaotic sea and (**b**) stability islands.

In order to better illustrate this tendency, we also perform another test by taking a *K* value where the stability islands and chaotic sea coexist, i.e., the case *K* = 2. For this case, it is evident from Fig. 2 that the region of chaotic sea with large positive Lyapunov exponents and the region of stability islands with Lyapunov exponents close to zero can easily be detected. This means that the system is ergodic within some portion of the phase space, whereas it is indeed non-ergodic within some other portion. Therefore we can check our previous findings using these portions separately. If we use initial conditions all taken from the portion where the system is ergodic (non-ergodic), we expect to see the same distribution function we have found before, namely the Gaussian (*q*-Gaussian with *q* = 1.935). In fact, this is exactly what we see in Fig. 6, which nicely corroborates our results given in Figs 3 and 4.

Finally we will be interested in another interesting question: what happens to the probability distribution if we do not take the portions of the phase space separately where the system is ergodic and non-ergodic but consider initial conditions coming from the whole phase space in the calculation of the probability distribution. This is really worth analysing since in this case one would expect a competition between initial conditions coming from the regions of the available phase space where the system is ergodic and non-ergodic and therefore between Gaussian and *q*-Gaussian behaviour. Needless to say, as the region in the phase space where the system is non-ergodic diminishes (like the case *K* = 10), Gaussian distribution will win, whereas the winner will be *q*-Gaussian as the region where the system is ergodic shrinks (like the case *K* = 0.2). We notice that, if these regions coexist, then this competition between Gaussian and *q*-Gaussian can be modelled as


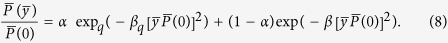


**Figure 7:**
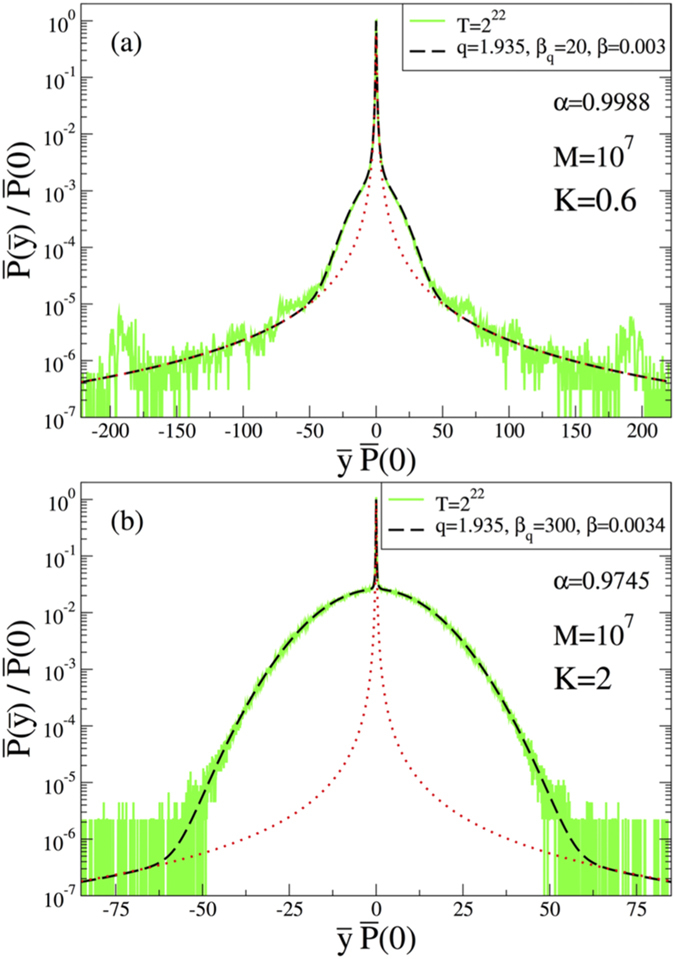
Normalized probability distribution function for the cases (**a**) *K* = 0.6 and (**b**) *K* = 2. In the calculations, initial conditions are randomly taken from the whole available phase space. In both cases, dotted red lines are the related *q*-Gaussian distributions.

We check this hypothesis using our two appropriate cases, namely, *K* = 0.6 and *K* = 2. The results are given in Fig. 7, where a corroboration can be seen at different scales; *α* decreases with increasing *K*, which in turn makes the phase-space ratio [number of points with Lyapunov exponent *λ* below *λ*_*threshold*_]/[number of points with *λ* above *λ*_*threshold*_] to decrease; for fixed *K*, this ratio increases with increasing *λ*_*threshold*_, but remains almost constant for 5 × 10^−5^ < *λ*_*threshold*_ < 10^−2^. The precise relation between *α* and this ratio remains to be studied but it is out of the scope of this manuscript and will be addressed elsewhere. It is easily seen that, as the stability islands dominate the whole phase space, the dominant distribution is the *q*-Gaussian with *q* = 1.935 (case *K* = 0.6), whereas the dominant one becomes the Gaussian if the chaotic sea invades more and more the whole phase space (case *K* = 2). It is in fact a very interesting result since, although for some portion of the phase space the system is ergodic, the signature of the *q*-Gaussian seems not to be erased even for very large *T* values. Therefore, apparently, for these two different portions of the phase space, the system behaves differently and each one is as robust as the other one. This mixture of Boltzmann-Gibbs statistical behaviour with Tsallis statistical behaviour is somewhat reminiscent of what was observed in a quite different system, namely one where overdamped motion is present^33^.

## Discussion

The phase space of the standard map presents regions of positive Lyapunov exponents coexisting with regions of zero Lyapunov exponents. The positive Lyapunov regions present mixing and thus the system is ergodic in those regions. For sufficiently low values of the control parameter *K*, the phase space is almost entirely dominated by zero Lyapunov behaviour and the distributions (obtained through time averaging, along the lines of central limit theorems) are *q*-Gaussians. As the value of *K* increases the measure of the zero Lyapunov regions decreases, and we see a continuous crossing, expressed by the parameter *α* in equation (8), between *q*-Gaussian distributions (Tsallis statistics) and Gaussian ones (Boltzmann-Gibbs statistics) with *β*_*q*_ → ∞ and *β* → *π* as *K* → ∞ (that is equivalent to *α* → 0). Remarkably enough, the distributions originated from initial conditions taken inside the region of islands, instead of over the entire phase space, yield one and the same value *q* = 1.935, independently on whether we consider one or many of these regions, and independently from *K*. Initial conditions taken within the chaotic sea always yield Gaussians with *β* = *π*. The variance of *q*-Gaussians with 5/3 < *q* < 3 diverges, though they have finite width. The *N*-fold convolution product of independent (or quasi-independent) *q*-Gaussians would asymptotically yield Lévy distributions^27^. Figure 5 neatly shows that this is *not* the case for the standard map: indeed, the actual time-averaging involves strong correlations. As previously discussed, since the standard map can be considered as a basic model for several physical systems (already mentioned in the Introduction), the present results are expected to be valid and useful for the statistical mechanical analysis of diverse phenomena, experimentally and observationally detected.

## Additional Information

**How to cite this article**: Tirnakli, U. and Borges, E. P. The standard map: From Boltzmann-Gibbs statistics to Tsallis statistics. *Sci. Rep.* **6**, 23644; doi: 10.1038/srep23644 (2016).
